# The Pathogenicity of *Shewanella algae* and Ability to Tolerate a Wide Range of Temperatures and Salinities

**DOI:** 10.1155/2018/6976897

**Published:** 2018-09-27

**Authors:** Shu-Ying Tseng, Po-Yu Liu, Yi-Hsuan Lee, Zong-Yen Wu, Chiu-Chen Huang, Ching-Chang Cheng, Kwong-Chung Tung

**Affiliations:** ^1^Department of Veterinary Medicine, College of Veterinary Medicine, National Chung-Hsing University, Taichung, Taiwan; ^2^Division of Infectious Diseases, Department of Internal Medicine, Taichung Veterans General Hospital, Taichung, Taiwan; ^3^Sing-Wang of Animal Hospital, Taichung, Taiwan; ^4^Laboratory Animal Service Center, Office of Research and Development, China Medical University, Taichung 40402, Taiwan

## Abstract

*Shewanella algae* is a rod-shaped Gram-negative marine bacterium frequently found in nonhuman sources such as aquatic ecosystems and has been shown to be the pathogenic agent in various clinical cases due to the ingestion of raw seafood. The results of this study showed that *S. algae* was present in approximately one in four samples, including water and shellfish samples. Positive reactions (API systems) in *S. algae* strains were seen for gelatinase (gelatin); however, negative reactions were found for indole production (tryptophan). *S. algae* is adapted to a wide range of temperatures (4°C, 25°C, 37°C, and 42°C) and salinity. Temperature is a key parameter in the pathogenicity of *S. algae* as it appears to induce hemolysis at 25°C and 37°C. *S. algae* exhibits pathogenic characteristics at widely varying temperatures, which suggests that it may have the ability to adapt to climate change.

## 1. Introduction

Recent studies indicate that climate change is driving ocean systems to recent increases in sea temperatures, with an associated risk of bacterial pathogens activity [1]. *Shewanella algae* has been identified as a new bacterial species, *Shewanella* spp., from clinical samples [2]. It is a rare human pathogen and symptoms of infection are often misidentified as *Vibrio* spp. [3]. It can be isolated from a wide range of environments, including fresh water, estuary, and the deep sea [4]. Risk factors associated with *S. algae* infections include chronic skin ulcer, chronic liver disease, and immune system disorders [5–7]. It appears to be more virulent in comparison with other *Shewanella* species [8–10].

Reports of infection with *S. algae* species in human cases are increasing, especially during the summer months and in tropical areas, such as India, China, and Taiwan [11–14]. In general, *S. algae* can be considered an opportunistic pathogen in humans exposed to a marine environment when it infects people via an existing soft tissue ulcer [15–22]. They have also been implicated in ear infection [23], eye infection, infective arthritis, osteomyelitis, bacteremia [24], infective endocarditis, and peritonitis in clinics [7]. Furthermore, *S. algae* infection tends to be associated with the ingestion of raw seafood, especially in individuals with hepatobiliary disease [3, 6, 13, 25]. This is a particular concern in some Asian regions in which there is a high demand for a wide variety of raw seafood. However, to date, there are few detailed data on *S. algae* with respect to its biochemical profiles and sources of infection in aquaculture.

In light of these questions, we conducted a study to analyze aquaculture and diverse water sources in order to determine the distribution of *S. algae*. Furthermore, we determined the profiles of samples obtained from diverse ranges of salinity and temperature. These results may serve as the basis of further study and could shed light on the ability of this pathogen to adapt to climate change.

## 2. Materials and Methods

### 2.1. Sample Collection and Preparation

Aquaculture and water samples were randomly collected from commercial oyster seedbeds along the west coast of Taiwan, fish markets, fishing ports, commercial abalone farms on the east coast, and some estuaries. The aquaculture samples were placed in sterile plastic bags, and water samples were collected in transportation tubes.

**Figure 1:**
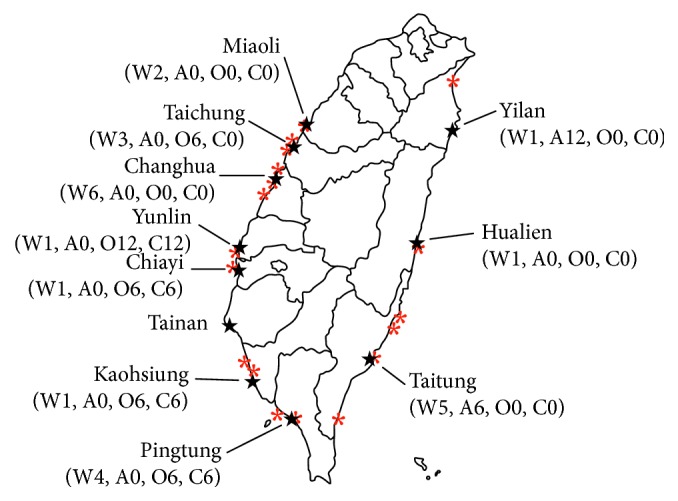
Location of sampling points of the aquaculture samples (★; A: Abalone; C: Clams; O: Oyster) and water samples (∗; W: water samples) in this study.

All samples were transported in refrigerated containers immediately after being collected. A total of 109 samples (water isolates (*n*=25) and aquaculture isolates (*n*=84) collected from 2012 to 2013 were investigated in this study (Figure 1).

Each isolate from the digestive glands of oysters, abalone, clams, and water samples were prepared on marine broth 2216 (MB; BD) as tenfold dilutions [26].

Secondary enrichment incubation was applied for 48 hours, and then 2 *μ*L of culture media was taken by loop and directly placed on the surface of marine agar 2216 (MA; BD). Colonies on marine agar were 2.0–2.5 mm in diameter, circular, convex with entire margins, and smooth after 2 days' incubation at 30°C. Orange-yellow or pink colonies on marine agar (BD) were identified as Gram-negative by Gram staining.

### 2.2. Biochemical and Nucleotide Sequence Analyses

The isolates were identified to species level by 16S rDNA sequence nucleotide sequence analyses. Each of the isolates identified by PCR analyses tested positive for 16S rDNA. Biochemical testing for phenotype was performed using an API20 NE (bioMérieux). All tests were performed according to the manufacturers' instructions. PCR-mediated amplification of the 16S rDNA was performed for confirmation of species identity. For nucleotide sequence analyses, genomic DNA was purified from overnight cultures of the isolates after growth on marine agar. The nucleotide was purified by using a QIAquick PCR purification kit (Qiagen). The extracted DNA was stored at −20°C until processing.

The remaining PCR solution was prepared for sequencing to confirm species identity. A fragment of the 16S rDNA gene was PCR-amplified from each genomic preparation using forward primer 27F: 5′-AGAGTTTGATCCTGGCTCAG-3′ and 1492R: 5′-TACGGCTACCTTGTTACGACTT-3ʹ. Reaction mixtures were incubated in an Eppendorf of Perkin-Elmerk GeneAmp 9600 PCR system. The reaction mix was put through the following temperatures with an initial denaturation for 1 min at 94°C, 1 min at 55°C, and 5 min at 72°C, for 30 cycles. The PCR products were thereafter cooled at 4°C. Sequences of these amplicons were completed by ABI 3730xl DNA Analyzer (Applied Biosystems). Reference sequences utilized in phylogenetic analysis were retrieved from NCBI's GenBank database. The 16S rDNA sequence data were compared with all currently available sequences of organisms belonging to the genus *Shewanella*.

Phenotypic characteristic assays included growth conditions (temperature and salinity tolerance). Assessment of biochemical features included measurement of oxidase, hydrogen sulfide, and indole production. Carbohydrate and fatty acids utilization, as well as hemolytic activity, was analyzed.

### 2.3. Phenotypic Characteristic Assays

Include growth conditions (temperature and salinity tolerance) and biochemical features (oxidase, hydrogen sulfide, and indole production; carbohydrate and fatty acids utilization; and hemolytic activity).

### 2.4. Cellular Characterization

The isolates were then grown in an overnight marine broth and the turbidity diluted to match a 0.5 MacFarland standard prior to inoculation at different temperatures (24 hrs∼7 days). All strains were tested for the ability to grow on MB and then placed into four separate incubators at 4°C, 25°C, 37°C, and 42°C for culturing (7 days). The growth of the isolates was routinely assessed indirectly by measuring the turbidity (OD_600nm_) using a UV-visible spectrophotometer (Tecan infinite 200, Switzerland). Growth was determined as an absorbance reading at or above 0.1.

### 2.5. Hemolysis Assay

To investigate the presence of potential virulence factors, we observed the hemolytic activity of *S. algae* on plates of 5% sheep blood agar (Commercialized Blood Agar Plate, Creative Co., Ltd., Taiwan) after incubation at two different temperatures (25°C and 37°C) and for two different times (24 hrs and 72 hrs).

### 2.6. Salinity Tolerance Assay

The salinity tolerance screening assay of the selected bacterial strains was carried out using tryptic soy broth (TSB, Difco) medium with 0–10% (w/v) concentration of NaCl. The flasks were inoculated with bacterial culture and incubated at 30°C on a rotator shaker (180 rpm) for 48 hrs. The bacterial growth assessment was carried out by measuring the turbidity (OD_600nm_) using a fluorescence spectrophotometer (Tecan infinite 200, Switzerland). The experiments were conducted in triplicate and the average values were recorded. The bacterial isolates were grown at 30°C for 7 days.

### 2.7. Statistical Analysis

Data were entered into Microsoft Excel 2017 (Microsoft Corporation, Redmond, USA) and analyzed.

## 3. Results

### 3.1. Quantity of Bacteria in Collected Samples

A total of 109 samples were collected. In total, 23% (19/84) of isolates from shellfishes and 28% (7/25) of water isolates were identified as *Shewanella algae* (Tables 1 and 2). We tested the significant differences in the isolation rates between water samples and shellfishes using Pearson's chi square with Yates' continuity correction. We found no significant difference between the two group (*p*=0.798 and *R*=0.022). The phenomenon suggests potential extensive water contamination which warrants continuous surveillance.

The bivalve mussels were identified by the Department of Life Sciences, National Chung Hsing University. The mussels were confirmed to be related to *Crassostrea angulata*, *Meretrix lusoria*, *Perna viridis*, *Geloina erosa*, and *Haliotis diversicolor*. Among these, the isolation rates of *Shewanella algae* were 2/18 in abalone (*Haliotis diversicolor*), 11/36 in oyster (*Crassostrea angulata*), and 6/30 in clams including *Meretrix lusoria*, *Perna viridis*, and *Geloina erosa* (Table 1). In addition, the locations with the greatest prevalence of *Shewanella algae* in water samples were commercial aquaculture farms on the west coast, with an isolation rate of 37.5% (3/8), followed by fish markets in fishing ports on the east coast, with a rate of 26.7% (4/15) (Table 2).

### 3.2. Characterization of Shewanella Strains

**Figure 2:**
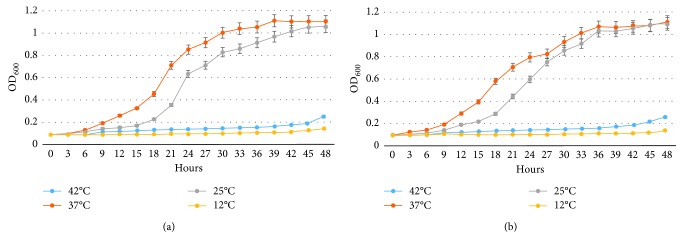
Growth curve of *Shewanella algae* isolates. (a) Aquaculture-origin, laboratory ID: O12. (b) Water-origin, laboratory ID: E-W1. *Shewanella algae* isolates were cultivated in LB broth at 12, 25, 37, and 42°C with shaking at 200 rpm. The optical density (OD_600_) was measured every 3 hours from zero point until 48 hours.

*S. algae* isolates were cultured at four different temperatures to establish reference data for future research on possible adaptation to global warming. The results showed *S. algae* isolates grew at three temperatures within the linear range (25, 37, and 42°C), but grew poorly at 4°C (Table 3). The growth curves of *S. algae* under different temperature are shown in Figure 2.

The biochemical profiles showed that all of the strains were unable to utilize some carbohydrates, but produced hydrogen sulfide (H_2_S). Positive reactions in *S. algae* strains were seen for H_2_S (from sodium thiosulfate) and cytochrome oxidase (oxidase test) and gelatinase (gelatin); however, negative results were found for indole production (tryptophan) and carbohydrates utilization, including arabinose, mannose, mannitol, adipic acid, and phenylacetic acid (Table 3).

Some *S. algae* isolates produced urease, which were related to positive urea reaction. Furthermore, most *S. algae* strains shared the ability to react with *N*-acetyl-glucosamine (NAG) as membrane substrates and reduction of nitrate to nitrite (potassium nitrate), and few *S. algae* isolates were able to assimilate maltose. Moreover, the majority of *S. algae* isolates assimilated capric acid and malic acid.

### 3.3. Effect of Salinity and Hemolysis In Vitro

The cultures were incubated at 30°C and all of them grew in the presence of a wide range of NaCl concentrations from 0, 2%, 6%, and 10% (w/v) (Table 3). Comparing the growth effects in different conditions, the isolates from aquaculture and water samples were favored by 0%, 2%, and 6% salinity. However, no bacterial growth was found at 10% NaCl.

Hemolysis occurred in sheep blood agar after incubation at two different temperatures (25°C and 37°C) (Table 3). One hundred percent hemolysis was found in *S. algae* from both aquaculture and water isolates at 37°C (after 72 hours). Compared with the 25°C group, only 50% hemolysis occurred after 72 hours.

## 4. Discussion

In this study, we investigated the prevalence of *S. algae* in a variety of environments around Taiwan. We collected 109 samples and identified *S. algae* in 23% (19/84) of isolates from shellfishes and in 28% (7/25) of water isolates (Tables 1 and 2).

*S. algae* can be found in samples from coastal areas, aquaculture farms, and aquaculture products [27]. *S. algae* is frequently found in the marine environment and is widely distributed in nature. Reports of infections with this opportunistic pathogen in humans are rare, although they are on the rise. In many clinical reports of hepatobiliary disease involving *S. algae* infection, there was a history of raw seafood ingestion [3, 25]. However, reports of *S. algae* infection in aquatic animals are rare. In view of this, we searched for articles on relevant cases in the ScienceDirect, PubMed, and Google Scholar databases using the following terms: “Aquaculture disease or Aquaculture products or Environment” in conjunction with “*Shewanella algae* or *Shewanella alga*.” The collected studies included research articles and case reports, as well as retrospective and series studies.  Table 4 shows a list of results for *S. algae* infections in aquaculture animals from 1999 to 2017 in different countries [15, 16, 28–40]. The results showed that *S. algae* is endemic in Asia. This finding is consistent with a number of studies conducted in areas with warm climates, largely in Asia [9, 41, 42]. A literature review of the period 1999 to 2017 showed that over 64% (9/14) of infection cases in aquatic animals were in Asia, including China, Japan, Malaysia, and Iran, as shown in Table 4. In addition, sea water was the predominant source of contamination and some cases were without disease symptoms.

In many reports of human infection with *S. algae*, the patient had a previous history of hepatobiliary disease or hemochromatosis and had recently consumed raw seafood [25, 28]. It is well understood that patients with hereditary hemochromatosis or hepatobiliary disease are prone to iron overload [43, 44]. *S. algae* could be tolerant to bile salts and may produce tetrodotoxin [42], exoenzymes, or siderophores [8], which are considered virulence factors. Furthermore, iron (Fe) serves as a terminal electron acceptor when *Shewanella* spp. are exposed to anoxic conditions [45, 46].

Our results showed *S. algae* strains are capable of growing in the presence of 0∼6% NaCl. *Shewanella* spp. are commonly found in marine environments and are believed to be halophilic bacteria [47, 48]. The traditional methods of processing seafood often take advantage of the preservative properties of salt, which permit long-term storage. The high salinity in the seafood product may influence the osmotic pressure and physiological properties of any bacteria that may be present. High salinity can result in the loss of microbial activity and cell plasmolysis. However, moderately salt-tolerant bacteria can resist or reduce the damaging effects of salt concentrations of up to 5-20% salinity [49]. Therefore, these salt-tolerant bacteria are potential food-borne pathogens. In our results, growth of *S. algae* was observed in a wide range of salinities (Table 3). Surprisingly, *S. algae* was also found in fresh water and nonmarine environments, and thus did not appear to require Na^+^, as shown in Table 3.

Furthermore, the seasonal growth and infection rate of *S. algae* peak during the summer. Based on the growth curves in our study, *S. algae* adapt to a wide range of temperatures, with optimal growth temperatures ranging at room temperature; under experimental conditions, the optimal temperatures for bacterial growth were 25°C and 37°C (Table 3). Comparing the two isolates of the curves based on optical density, the curves of growth rate versus temperature are in direct proportion as drawn in Figure 2. This probably explains why reports of *S. algae* infection are more common in warm water areas or tropical regions during the summer than in cold-water environments [8]. Prior study revealed that *S. algae* grows under the condition of temperature 26–34°C, pH 5–9 [40]. The data provide further support of the capacity of survival of *S. algae* under ocean acidification caused by global warming.

Earlier research has suggested that hemolysis may be used as a marker to predict potentially virulent strains of *S. algae* [3]. We previously reported that a substantial number of *S. algae* strains were capable of growing on sheep blood agar. *S. algae* strains from all isolates exhibited hemolysis on sheep blood agar. In Table 3, it can be seen hemolytic activity on sheep blood agar was high (90% to 100%) at 37°C. In contrast, there was no obvious hemolysis on the first day of incubation at 25°C; however, it was clearly evident on the second day. This finding implies that *Shewanella* species exert various forms of hemolysis. Our results are also consistent with epidemiologic studies in Denmark [28] and Taiwan [25], which showed the infection rates of *S. algae* were correlated with temperature fluctuation. In general, most *S. alga* strains exhibited hemolysis after prolonged incubation (48 to 72 h) and the area of hemolysis was clear.

The major characteristics in all *S. algae* isolates in this study include the ability to exert a strong hemolytic effect: an inability to utilize carbohydrates, although a few isolates were able to use maltose from some water samples. Other studies previously found that a few *Shewanella* species could utilize L-arabinose and glucose [9, 50]; however, we were not able to confirm these results.

*S. algae* utilizes few carbohydrates as the sole carbon source according to the results of this study. In principle, bacteria are quite diverse in terms of nutrient utilization and metabolic requirements in a specific environment, owing to their biosynthetic capabilities. Previous biochemical characterization studies have suggested that while some *Shewanella* species are able to metabolize different sugars for growth, they might be limited to biosynthetic purposes such as cell wall synthesis or as a storage molecule, rather than as a carbon source [51, 52]. This could explain the apparent inconsistencies among several experimental observations. In our results, only 14% of *S. algae* from diverse water sources and 16% of aquaculture isolates utilized maltose (Table 3). From these, we may infer *S. algae* adapt to environmental changes via different biosynthetic pathways. *Shewanella* spp. possess a number of mechanisms to assimilate carbohydrates from the environment. It has been proposed that *Shewanella oneidensis* MR-1 uses the formaldehyde produced from pyruvate during growth under anaerobic or oxygen-limited conditions [53].

In addition, the results of indole production were all negative for *S. algae* isolates. Indole can act as an extracellular signal to regulate biofilm-promoting factors and the expression of adhesion molecules [54]. Bacteria which give negative results for the indole test include some *Aeromonas* species and *Vibrio* spp. [55]. Recent studies showing that non-indole-producing bacteria generate various oxygenases which may degrade indole or interfere with indole signaling [56, 57]. Many oxygenase and reductase reactions may be involved in metal ion facilitation in bacteria [58]. The ability of *S. oneidensis* to reduce oxidized metals or nitrate effectively has been identified as an important intrinsic activity of *Shewanella* species [59–62]. These results all indicate that *S. algae* is capable of adapting to environmental changes. Further studies are needed to clarify the role of regulating the intrinsic activity of S*. algae*.

There were several limitations in this study. First, continuous multisite surveillance is needed to demonstrate seasonal variation and long-term effect of global warming on *S. algae* population. Second, the lack of discrimination by the API systems for *Shewanella* bacteria is understandable since the isolates show diverse results in biochemical testing. It is necessary to improve identification schemes to identify pathogenic and nonpathogenic strains of *S. algae* in the natural environment.

In summary, our study identified the presence of *S. algae* in water and aquaculture products in Taiwan. We further identified hemolytic activity in all isolates, indicating that this species of bacteria possesses a pathogenic potential. We found high levels of *S. algae* isolates contained in diverse sources (oysters, abalone, clam, and water samples). The ability of *Shewanella* strains to tolerate a wide range of temperatures and salinities in experimental challenges may be due to the expression or repression of genes, but further research is needed to explore the potential underlying mechanisms involved. These results suggest that monitoring the levels of pathogenic species and strains should be continued in Taiwan and expanded to other tropical and subtropical zones in Asia.

## Figures and Tables

**Table 1:** Incidence of *Shewanella algae* in aquaculture samples.

Location	Sources of sample	Genus species	Total no. of samples	No. yielding *Shewanella algae*
TaiTung	Abalone (cultured)	*Haliotis diversicolor*	6	2
Yilan	Abalone (cultured)	*Haliotis diversicolor*	12	0
YunLin	Oyster (cultured)	*Crassostrea angulate*	12	1
ChiaYi	Oyster (cultured)	*Crassostrea angulate*	6	2
KaoHsiung	Oyster (cultured)	*Crassostrea angulate*	6	2
Taichung	Oyster (Fish market)	*Crassostrea angulate*	6	2
PingTung	Oyster (cultured)	*Crassostrea angulate*	6	4
YunLin	Clam (cultured)	*Geloina erosa*	12	1
KaoHsiung	Clam (cultured)	*Meretrix lusoria*	6	3
ChiaYi	Clam (cultured)	*Meretrix lusoria*	6	0
PingTung	Clam (cultured)	*Perna viridis*	6	2
*Total number*			84	19
*Isolation rate * **** *(%)*				23

**Table 2:** Occurrence of *Shewanella algae* in different water-sampling sites.

Location	Sources of sample	No. of cultured	No. yielding *Shewanella algae*
Miaoli County (Houlong Township)	Mariculture	2	1
Changhua County (Yuanlin Township)	Sea gate	3	1
Changhua County (Fishing port)	Sea water	3	0
Taichung City (Dali Dist.)	Fresh water	1	0
Taichung City (Wuqi Dist.)	Sea water	2	0
Yunlin County (Kouhu Township)	Mariculture	1	0
Chiayi County (Budai Township)	Sea water	1	0
Kaohsiung City (Ziguan Dist.)	Sea water	1	0
Pingtung County (Fangliao Township)	Mariculture	4	2
Taitung County	Sea water	4	2
Taitung County (Donghe Township)	Fresh water	1	0
Hualien County (Fengbin Township)	Sea water	1	1
Yilan County (Toucheng Township)	Mariculture	1	0
Total numbers		25	7
Isolation rate (%)			28

**Table 3:** Phenotypic characteristics of *Shewanella algae* isolates.

Reaction	Values are positive percentages	
	Aquaculture isolates (*n*=19) (%)	Water isolates (*n*=7) (%)
*Growth at*		
4°C on MB	0	14
25°C on MB	100	100
37°C on MB	100	100
42°C on MB	74	100
30°C in LB with 0% NaCl	100	100
30°C in LB with 2% NaCl	100	100
30°C in LB with 6% NaCl	100	100
30°C in LB with 10% NaCl	0	0

*Hemolysis of blood agar plate at*		
37°C (24 hrs)	89	100
25°C (24 hrs)	0	0
37°C (72 hrs)	100	100
25°C (72 hrs)	53	57

*Reactions/enzymes (API20NE)*		
Reduction of nitrates to nitrites	100	100
Indole production	0	0
Glucose fermentation	0	0
Arginine dihydrolase	5	0
Urease	32	29
*β*-Glucosidase	53	43
Gelatinase	95	100
*β*-Galactosidase	11	0

*Assimilation (API20NE)*		
Glucose	0	14
Arabinose	0	0
Mannose	0	0
Mannitol	0	0
*N*-Acetyl-glucosamine	95	100
Maltose	16	14
Potassium gluconate	0	14
Capric acid	79	71
Adipic acid	0	0
Malic acid	100	86
Trisodium citrate	11	29
Phenylacetic acid	0	0

*Others*		
Oxidase	100	100
H_2_S-production (TSIA)	100	100

**Table 4:** Distribution of *Shewanella algae* in environment samples and aquaculture animals (1999–2017).

Year	Region	Sampling location	Host	Disease symptoms	References
1999	Denmark	Sea water	Environment samples	No	Gram et al., [28]
2000	Denmark	Sea water	Environment samples	No	Vogel et al., [15]
2002	China	Sea water	Scinenops ocellata	Ulcer disease	Chang et al., [16]
2006	China	Pond water	Abalone	Whitening, shrunken muscles	Cai et al., [29]
2008	USA	Sea water	Shellfish	Nonavailable	Richards et al., [30]
2009	Japan	Sea water	Sea cucumber	Nonavailable	Beleneva et al., [31]
2010	Malaysia	Tank water	Shrimp	Healthy post larvae	Zadeh et al., [32]
2010	Japan	Tank water	Pufferfish	Healthy fish	Sugita et al., [33]
2011	USA	Sea water	Sediments	No	Cummings et al., [34]
2012	China	Sea water	Environment samples	No	Zhao and Dang, [35]
2013	China	Sea water	Marine culture	No	Liu et al., [25, 36, 37]
2013	China	Sea water	Deep-sea sediments	No	Jiang et al., [36]
2013	Portuguese	Sea water	Deep sea	No	Martins et al., [38]
2015	Iran	Sea water	Mussels/sediment	No	Bayat et al., [39]
2017	China	Sea water	Fish	Noticeable histological lesions	Z. Han et al., [40]

## Data Availability

The data used to support the findings of this study are currently under embargo while the research findings are published. Requests for data, 6 months after publication of this article, will be considered by the corresponding author.

## References

[1] Bally M., Garrabou J. (2007). Thermodependent bacterial pathogens and mass mortalities in temperate benthic communities: a new case of emerging disease linked to climate change. *Global Change Biology*.

[2] Nozue H., Hayashi T., Hashimoto Y. (1992). Isolation and characterization of *Shewanella alga* from human clinical specimens and emendation of the description of *S. alga* Simidu et al., 1990, 335. *International Journal of Systematic Bacteriology*.

[3] Myung D. S., Jung Y.-S., Kang S.-J. (2009). Primary *Shewanella algae* bacteremia mimicking *Vibrio* septicemia. *Journal of Korean Medical Science*.

[4] Fu X., Wang D., Yin X., Du P., Kan B. (2014). Time course transcriptome changes in *Shewanella algae* in response to salt stress. *PLoS One*.

[5] Domínguez H., Vogel B. F., Gram L., Hoffmann S., Schaebel S. (1996). *Shewanella alga* bacteremia in two patients with lower leg ulcers. *Clinical Infectious Diseases*.

[6] Otsuka T., Noda T., Noguchi A., Nakamura H., Ibaraki K., Yamaoka K. (2007). *Shewanella* infection in decompensated liver disease: a septic case. *Journal of Gastroenterology*.

[7] Srinivas J., Pillai M., Vinod V., Dinesh R. K. (2015). Skin and soft tissue infections due to *Shewanella algae*: an emerging pathogen. *Journal of Clinical and Diagnostic Research*.

[8] Khashe S., Janda J. M. (1998). Biochemical and pathogenic properties of *Shewanella alga* and *Shewanella putrefaciens*. *Journal of Clinical Microbiology*.

[9] Holt H. M., Gahrn-Hansen B., Bruun B. (2005). *Shewanella algae* and *Shewanella putrefaciens*: clinical and microbiological characteristics. *Clinical Microbiology and Infection*.

[10] Janda J. M. (2014). *Shewanella*: a marine pathogen as an emerging cause of human disease. *Clinical Microbiology Newsletter*.

[11] Tsai M.-S., You H.-L., Tang Y.-F., Liu J.-W. (2008). *Shewanella* soft tissue infection: case report and literature review. *International Journal of Infectious Diseases*.

[12] Liu P.-Y., Shi Z. Y., Lin C. F. (2012). *Shewanella* infection of snake bites: a twelve-year retrospective study. *Clinics*.

[13] Kim B. K., Cho S.-Y., Kang B. (2014). A case of spontaneous bacterial peritonitis with bacteremia caused by *Shewanella algae*. *Infection & Chemotherapy*.

[14] Sumathi B. G., Kumarswamy S. R., Amritam U., Arjunan R. (2014). *Shewanella algae*: first case report of the fast emerging marine pathogen from squamous cell carcinoma patient in India. *South Asian Journal of Cancer*.

[15] Vogel B. F., Holt H. M., Gerner-Smidt P., Bundvad A., Søgaard P., Gram L. (2000). Homogeneity of danish environmental and clinical isolates of *Shewanella algae*. *Applied and Environmental Microbiology*.

[16] Chang C., Chaoqun H., Xiaoyan C., Luping Z. (2002). Identification and characterization of *Shewanella algae* as a novel pathogen of ulcer disease of fish *Scinenops ocellata*. *Oceanologia et Limnologia Sinica*.

[17] Wang T. (2009). Model of life expectancy of chronic hepatitis B carriers in an endemic region. *Journal of Epidemiology*.

[18] Sharma K. K., Kalawat U. (2010). Emerging infections: *Shewanella*—a series of five cases. *Journal of Laboratory Physicians*.

[19] Nath R., Choudhury G., Saikia L., Das P. (2011). Isolation of *Shewanella algae* from rectal swabs of patients with bloody diarrhoea. *Indian Journal of Medical Microbiology*.

[20] Wait S., Chen D.-S. (2012). Towards the eradication of hepatitis B in Taiwan. *Kaohsiung Journal of Medical Sciences*.

[21] Wagner N., Otto L., Podda M., Schmitt Y., Tappe D. (2013). Travel-related chronic hemorrhagic leg ulcer infection by *Shewanella algae*. *Journal of Travel Medicine*.

[22] Wu T.-W., Lin H. H., Wang L.-Y. (2013). Chronic hepatitis B infection in adolescents who received primary infantile vaccination. *Hepatology*.

[23] Holt H. M., Søgaard P., Gahrn-Hansen B. (1997). Ear infections with *Shewanella alga*: a bacteriologic, clinical and epidemiologic study of 67 cases. *Clinical Microbiology and Infection*.

[24] Iwata M., Tateda K., Matsumoto T., Furuya N., Mizuiri S., Yamaguchi K. (1999). Primary *Shewanella alga* Septicemia in a Patient on Hemodialysis. *Journal of Clinical Microbiology*.

[25] Liu P.-Y., Lin C.-F., Tung K.-C. (2013). Clinical and microbiological features of *Shewanella* Bacteremia in patients with hepatobiliary disease. *Internal Medicine*.

[26] Al-Harbi A. H., Naim Uddin M. (2004). Seasonal variation in the intestinal bacterial flora of hybrid tilapia (*Oreochromis niloticus×Oreochromis aureus*) cultured in earthen ponds in Saudi Arabia. *Aquaculture*.

[27] Cabral J. P. S. (2010). Water microbiology. Bacterial pathogens and water. *International Journal of Environmental Research*.

[28] Gram L., Bundvad A., Melchiorsen J., Johansen C., Vogel B. F. (1999). Occurrence of *Shewanella algae* in Danish coastal water and effects of water temperature and culture conditions on its survival. *Applied and Environmental Microbiology*.

[29] Cai J., Chen H., Thompson K. D., Li C. (2006). Isolation and identification of *Shewanella alga* and its pathogenic effects on post-larvae of abalone *Haliotis diversicolor supertexta*. *Journal of Fish Diseases*.

[30] Richards G. P., Watson M. A., Crane E. J., Burt I. G., Bushek D. (2008). *Shewanella* and *Photobacterium* spp. in oysters and seawater from the Delaware Bay. *Applied and Environmental Microbiology*.

[31] Beleneva I. A., Magarlamov T. Y., Eliseikina M. G., Zhukova N. V. (2009). Biochemical and pathogenic properties of the natural isolate of *Shewanella algae* from peter the great bay, Sea of Japan. *Journal of Invertebrate Pathology*.

[32] Zadeh S. S., Saad C. R., Christianus A. (2010). Assessment of growth condition for a candidate probiotic, *Shewanella algae*, isolated from digestive system of a healthy juvenile *Penaeus monodon*. *Aquaculture International*.

[33] Sugita H., Sugiyama K., Itoi S. (2010). Culturable bacterial flora in the intestinal tract of Japanese Pufferfish *Takifugu rubripes*. *Aquaculture Science*.

[34] Cummings D. E., Archer K. F., Arriola D. J. (2011). Broad dissemination of plasmid-mediated quinolone resistance genes in sediments of two urban coastal wetlands. *Environmental Science & Technology*.

[35] Zhao J., Dang H. (2012). Coastal seawater bacteria harbor a large reservoir of plasmid-mediated quinolone resistance determinants in Jiaozhou Bay, China. *Microbial Ecology*.

[36] Jiang W., Xia B., Liu Z. (2013). A serine hydroxymethyltransferase from marine bacterium *Shewanella algae*: isolation, purification, characterization and l-serine production. *Microbiological Research*.

[37] Liu G., Zhou J., Meng X. (2013). Decolorization of azo dyes by marine *Shewanella* strains under saline conditions. *Applied Microbiology and Biotechnology*.

[38] Martins A. (2013). Photoprotective bioactivity present in a unique marine bacteria collection from Portuguese deep sea hydrothermal vents. *Marine Drugs*.

[39] Bayat Z., Hassanshahian M., Hesni M. A. (2015). Enrichment and isolation of crude oil degrading bacteria from some mussels collected from the Persian Gulf. *Marine Pollution Bulletin*.

[40] Han Z., Sun J., Lv A. (2017). Isolation, identification and characterization of *Shewanella algae* from reared tongue sole, *Cynoglossus semilaevis* Günther. *Aquaculture*.

[41] Finkelstein R., Oren I. (2011). soft tissue infections caused by marine bacterial pathogens: epidemiology, diagnosis, and management. *Current Infectious Disease Reports*.

[42] Vignier N., Théodose R., Barreau M. (2013). Human infection with *Shewanella putrefaciens* and *S. algae*: report of 16 cases in martinique and review of the literature. *American Journal of Tropical Medicine and Hygiene*.

[43] Schamroth L., Edelstein W., Politzer W. M., Stevens N. (1956). Serum iron in the diagnosis of hepatobiliary disease. *BMJ*.

[44] Pietrangelo A. (2004). Hereditary hemochromatosis: a new look at an old disease. *New England Journal of Medicine*.

[45] Straub K. L., Benz M., Schink B. (2001). Iron metabolism in anoxic environments at near neutral pH. *FEMS Microbiology Ecology*.

[46] Weber K. A., Achenbach L. A., Coates J. D. (2006). Microorganisms pumping iron: anaerobic microbial iron oxidation and reduction. *Nature Reviews Microbiology*.

[47] DeFrank J. J., Beaudry W. T., Cheng T.-C., Harvey S. P., Stroup A. N., Szafraniec L. L. (1993). Screening of halophilic bacteria and *Alteromonas* species for organophosphorus hydrolyzing enzyme activity. *Chemico-Biological Interactions*.

[48] Gram L., Huss H. H. (1996). Microbiological spoilage of fish and fish products. *International Journal of Food Microbiology*.

[49] Larsen H. (1986). Halophilic and halotolerant microorganisms-an overview and historical perspective. *FEMS Microbiology Letters*.

[50] Vogel B. F., Jørgensen K., Christensen H., Olsen J. E., Gram L. (1997). Differentiation of *Shewanella putrefaciens* and *Shewanella alga* on the basis of whole-cell protein profiles, ribotyping, phenotypic characterization, and 16S rRNA gene sequence analysis. *Applied and Environmental Microbiology*.

[51] Venkateswaran K., Moser D. P., Dollhopf M. E. (1999). Polyphasic taxonomy of the genus *Shewanella* and description of *Shewanella oneidensis* sp. nov. *International Journal of Systematic Bacteriology*.

[52] Heidelberg J. F., Paulsen I. T., Nelson K. E. (2002). Genome sequence of the dissimilatory metal ion–reducing bacterium *Shewanella oneidensis*. *Nature Biotechnology*.

[53] Tang Y. J., Meadows A. L., Kirby J., Keasling J. D. (2007). Anaerobic central metabolic pathways in *Shewanella oneidensis* MR-1 reinterpreted in the light of isotopic metabolite labeling. *Journal of Bacteriology*.

[54] Martino P. D., Fursy R., Bret L., Sundararaju B., Phillips R. S. (2003). Indole can act as an extracellular signal to regulate biofilm formation of *Escherichia coli* and other indole-producing bacteria. *Canadian Journal of Microbiology*.

[55] Lee J.-H., Lee J. (2010). Indole as an intercellular signal in microbial communities. *FEMS Microbiology Reviews*.

[56] Hu M., Zhang C., Mu Y., Shen Q., Feng Y. (2010). Indole Affects biofilm formation in bacteria. *Indian Journal of Microbiology*.

[57] Han T. H., Lee J.-H., Cho M. H., Wood T. K., Lee J. (2011). Environmental factors affecting indole production in *Escherichia coli*. *Research in Microbiology*.

[58] Varkey A. J., Dlamini M. D., Mansuetus A. B., Tiruneh A. T. (2013). Germicidal action of some metals/metal ions in combating *E. coli* bacteria in relation to their electro-chemical properties. *Journal of Water Resource and Protection*.

[59] Richardson D. J. (2000). Bacterial respiration: a flexible process for a changing environment. *Microbiology*.

[60] DiChristina T. J., Moore C. M., Haller C. A. (2002). Dissimilatory Fe(III) and Mn(IV) reduction by *Shewanella putrefaciens* requires ferE, a homolog of the pulE (gspE) type II protein secretion gene. *Journal of Bacteriology*.

[61] Hau H. H., Gralnick J. A. (2007). Ecology and biotechnology of the genus *Shewanella*. *Annual Review of Microbiology*.

[62] Wen J., Zhou S., Chen J. (2014). Colorimetric detection of *Shewanella oneidensis* based on immunomagnetic capture and bacterial intrinsic peroxidase activity. *Scientific Reports*.

